# Delayed Urethral Obstruction after Migration of a Ballistic Pellet in an Alpine Wether

**DOI:** 10.1155/2023/3531856

**Published:** 2023-07-17

**Authors:** Joe S. Smith, Adrien-Maxence Hespel, Jessica D. Garcia, Krista L. Lipe, Stephanie A. Kleine, Pierre-Yves Mulon

**Affiliations:** ^1^Large Animal Clinical Sciences, College of Veterinary Medicine, University of Tennessee, Knoxville, TN, USA; ^2^Biomedical Sciences, College of Veterinary Medicine, Iowa State University, Ames, IA, USA; ^3^Small Animal Clinical Sciences, College of Veterinary Medicine, University of Tennessee, Knoxville, TN, USA

## Abstract

A one-year-old alpine wether was presented for emergency evaluation of stranguria. Diagnostics identified a moderately distended bladder and mild dehydration. Preliminary lateral radiographs identified two metallic structures consistent with projectile pellets in the pelvic and perineal regions and no evidence of radiopaque uroliths. A tube cystostomy was performed, and a contrast urethrogram revealed one of the pellets in the perineal region was in proximity to the urethral obstruction. Subsequent radiography and ultrasound identified the pellet as being within the lumen of the urethra. Examination of the trichotomized skin revealed two scars, including a scar over the paralumbar fossa in the region of the urinary bladder suggestive of a projectile injury. The pellet was removed by a perineal urethrotomy. The patient was able to spontaneously urinate after urethrotomy, passed a tube cystostomy challenge two weeks after surgery, and was discharged. No complications were reported. While uncommon in the veterinary and comparative medical literature, clinicians should consider the potential for projectile pellets to migrate into the urinary tract after initial injury.

## 1. Introduction

A one-year-old alpine wether presented to the University of Tennessee Charles and Julie Wharton Large Animal Hospital with stranguria and abdominal distension, which started 5 hours before transportation. His typical diet included grass hay with alfalfa pellets and commercial goat grain with access to pasture. No mineral supplementation or previous medical history was reported. The physical examination was within normal limits except for the palpation of urethral pulses on the digital rectal exam. Posturing to urinate in an unproductive fashion during the examination was noted. There was no evidence of recent trauma. Point-of-care bloodwork (electrolytes, renal values, packed cell volume, and total solids) was unremarkable other than moderate dehydration (packed cell volume 47%, total solids 7.0).

Ultrasound of the abdomen and pelvic region revealed a moderately distended bladder (10.75 cm in diameter). Right-lateral radiographs of the caudal abdomen and pelvis revealed no evidence of uroliths. Two radiopaque foreign bodies identified as ballistic projectiles were noticed in the pelvic region and presumed incidental. Routine radiographic emergency protocol for goats with suspected urinary blockages targets the presence of radiopaque uroliths in the caudal abdomen and the pelvic region with a unique lateral view; therefore, orthogonal radiographs were not acquired at the time of presentation. An ultrasound-guided decompressive cystocentesis was performed using a 20-gauge spinal needle (3.5 in) under sedation with midazolam (0.2 mg/kg) and butorphanol (0.2 mg/kg) administered intravenously. Approximately 1 liter of urine was removed from the bladder. The patient was medically stabilized for the rest of the night and was administered a booster vaccination for *Clostridium perfringens*, types C and D, tetanus (Bar-Vac CDT, Boehringer Ingelheim, Georgia, US), and procaine penicillin G (22,000 IU/kg subcutaneously).

No urination was observed overnight, and urinary obstruction was deemed to be still present the following morning. Therefore, routine tube cystostomy surgery was performed for urinary diversion to investigate the presence of nonradiopaque uroliths. One dose of flunixin meglumine (1.1 mg/kg IV) and a second dose of procaine penicillin G (22,000 IU/kg SC) were administered prior to surgery.

The wether was anesthetized with midazolam (0.2 mg/kg) and morphine (0.2 mg/kg) premedication, induction with ketamine (1.0 mg/kg) and midazolam (0.2 mg/kg), and maintenance with isoflurane in oxygen. As already noticed by the radiographic investigation, the performed digital sweep did not reveal stones within the urinary bladder. A rubber catheter was then placed into the urinary bladder and through the urethra in a normograde fashion. Attempts to flush sterile saline through the catheter into the urethra were unsuccessful.

**Figure 1:**
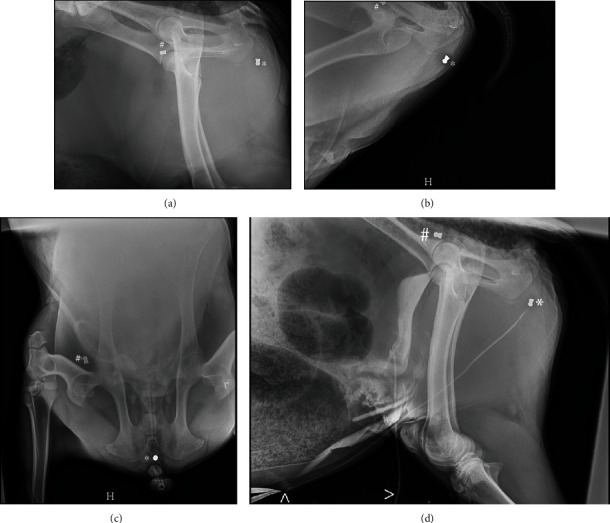
(Radiographs) Left lateral radiograph of the abdomen (a), left lateral radiograph of the abdomen with the hind limbs flexed forward (b), and ventrodorsal radiographs of the pelvis (c). Panels (a, b) were taken on initial intake, with panels (c, d) taken later during further workup. There are two pellets superimposed with the caudal abdomen on the lateral view. One pellet is noted superimposed with the thigh musculature of the right hind limb on the ventrodorsal projection (#). However, the second one remains in the plane of the urethra on all 3 projections (^∗^). Lateral radiograph of the abdomen. The pellets that were noted on the plain radiographs remain visible (# and ^∗^). A positive cystogram (d) was initially performed through the cystotomy tube (>). Subsequently, a urethrogram was performed (D), and the urethra is maintained clamped (^). The urethra is filled with positive contrast up to the point of the pellet (^∗^), but no contrast is extending past the urethra.

After the procedure, while the patient was under general anesthesia, contrast normograde and retrograde cystourethrograms were performed, revealing the obstruction to be in the patient's pelvic urethra (Figure 1). The two metal-opaque ballistic foreign bodies present on the preoperative radiographs were also identified during the cystourethrogram (Figure 1). One appeared to be located in proximity to the urethral obstruction. At this point, the decision was made to recover the wether from anesthesia due to the duration of the anesthetic event, despite the lack of urethral patency. Postoperative medical management included intravenous fluid therapy at approximately one and a half time maintenance. As differentials included a struvite (radiolucent) stone that would respond to urinary acidification, ammonium chloride was incorporated into the treatment regimen. Therapies included ammonium chloride (100 mg/kg PO, q 12 hr for 5 days), penicillin procaine G (22,000 IU/kg SC, q 12 h for 10 days), flunixin meglumine (1.1 mg/kg IV, once postoperatively), followed by meloxicam (0.5 mg/kg PO, q 24 hr for 13 days). The patient continued to drip urine from the cystostomy tube during the entire postoperative period, and the urine pH remained around 6.5.

**Figure 2:**
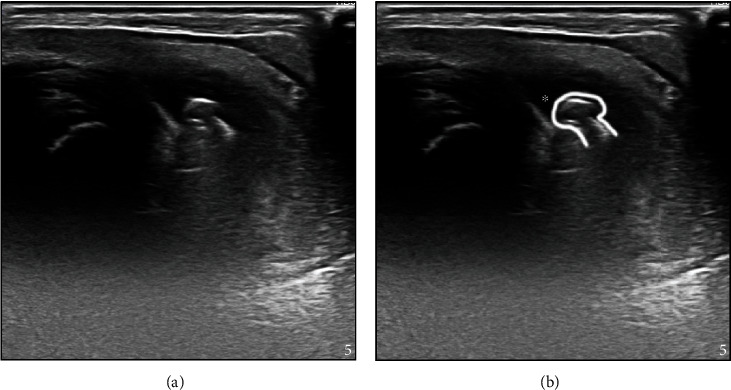
(Ultrasound) Longitudinal image of the perineal urethra ((a) nonannotated, (b) annotated image). The proximal aspect of the urethra is on the left of the image. On the annotated image (b), the urethra is moderately distended (^∗^) just proximal to the pellet (contoured).

**Figure 3:**
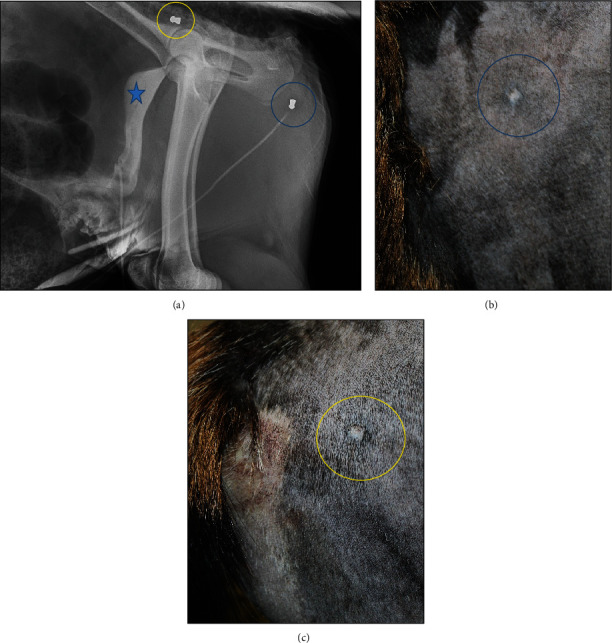
(Skin images and corresponding radiographic locations): (a) Lateral radiograph identifying pellet obstructing urethra (blue star), and pellet in the hip region (yellow circle). The blue circle in (a) corresponds to the scar noted in (b) over the paralumbar fossa. The scar highlighted in 3C is in the immediate region of the pellet in the hip region.

Two days after surgery and after further review of the patient's medical history with the team of veterinarians, it was determined that orthogonal radiographs and urethral ultrasound should be performed to ensure a full evaluation of the projectiles because the obstruction was still not resolved. One ballistic foreign body was identified in the distal pelvic urethra in the perineal region on both orthogonal radiographs and confirmed to be within the lumen of the urethra on ultrasound, with the second noted to be superficial in the hip region (Figures 1 and 2). In an attempt to locate the pellets' location of entry, the patient's flank and perineum were shaved. One scar was identified in proximity to the pellet over the hip, and another scar was identified in the paralumbar fossa (Figure 3, with corresponding radiographic locations). The goat's owners elected to remove the ballistic projectile obstructing the urethra.

**Figure 4:**
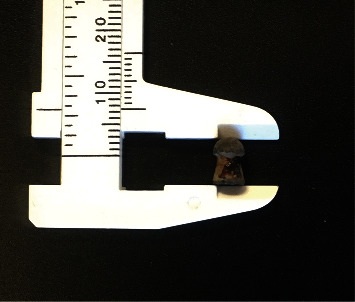
The projectile pellet was recovered from the urethra of the patient. Note the cone-shaped base and rounded tip, without evidence of deformation. The diameter (not pictured) was measured to be 4.5 mm.

The patient was anesthetized using the same protocol as reported for the tube cystostomy. Upon identification of the ballistic projectile on urethral ultrasound, sterile saline was used to flush retrogradely into the urethra in an attempt to locate the ballistic pellet either within or adjacent to the urethra. When flushed, the ballistic pellet moved retrogradely further within the pelvic urethra, thus confirming its location within rather than adjacent to the urethra. A 3 cm incision was made into the perineum. A blunt dissection was performed with Metzenbaum scissors to visualize the retractor penis muscle. An incision was made between the pair of retractor penis muscles to gain access to the penis. Sharp dissection was used to cut through the corpus spongiosum to expose the urethra. A mild hemorrhage was present upon incising the corpus spongiosum and was controlled with temporary compression with gauzes. The urethra was exposed, and a urethrotomy was performed using a #10 blade. Once opened, Kelly hemostatic forceps were used to grasp the 5F rubber urinary catheter placed into the urethra prior to surgery for the retrograde flushing. Using the Kelly hemostatic forceps, the pellet (Figure 4) was grasped and removed from the urethra. The rubber urinary catheter was introduced in the proximal portion of the urethra, and the urethra was closed with a cruciate pattern using 4-0 PDS using the catheter as a stent. Simple, interrupted sutures were placed in the gaps between the cruciates using the same suture material. The paired retractor penis muscles were apposed in a simple continuous pattern using 4-0 PDS for the distal portion of the incision and 2-0 PDS for the proximal portion of the incision. The subcutaneous layer was closed with a simple continuous pattern using 2-0 PDS. Skin staples were placed on the skin incision to close it.

An intraoperative rubber catheter was placed in a retrograde fashion into his urethra in an attempt to keep the urethra patent during postoperative urethral healing. The patient removed the catheter about 12 hours after surgery, and it was not replaced. The wether appeared uncomfortable postoperatively, so he was administered acepromazine (0.01 mg/kg IM, once) for mild sedation in an attempt to relax the retractor penis muscle. After the second surgery, the patient continued on the same medical management plan set after the initial surgery.

Fourteen days after the initial presentation, the patient underwent a tube challenge, with the Foley catheter placed during the tube cystostomy clamped and visualized to see if he was able to urinate out his urethra, as this would be the only urinary egress present. The patient passed the challenge and was seen urinating normally without straining. His foley catheter was deflated and removed. He was discharged 16 days after his initial presentation.

## 2. Discussion

Urethral obstruction is a common occurrence in domestic goats, especially in castrated males [1–3]. Multiple urinary obstruction cases have been described in the caprine literature, but almost all descriptions of these cases are of uroliths, including struvite, calcium carbonate, silica, and amorphous magnesium [1]. Urinary obstructions resulting from urethral strictures and masses have also been described [1]. In the literature, there is a paucity of descriptions of urethral obstructions secondary to foreign objects. Urinary obstruction due to bullet migration to the lower urinary tract has been described in human patients, and in the veterinary literature, one case report describes a urethral obstruction in a dog second to a gunshot [4–6]. To the author's knowledge, no cases describing urethral obstruction by ballistic projectiles in a large animal are reported in the literature.

Multiple techniques have been described for the management of urinary obstructions in goats including tube cystostomy, cystotomy, perineal urethrostomy, urethrotomy, bladder marsupialization, and vesiculoprepucial anastomosis [1, 7, 8]. In this case, tube cystostomy was selected before the intraluminal presence of the pellet was appreciated, as the technique would allow for urine to drain while the patient was managed. After the presence of the pellet in the urethral lumen was confirmed, a urethrotomy was performed to remove the pellet. Tube cystostomy, combined with urethrotomy, for the management of obstructive urolithiasis in goats has been demonstrated to have successful long-term outcomes [2, 7]. In this case, the implication of the ballistic foreign body present in the perineal region was not evident on the initial radiographic work-up. In the absence of radiopaque uroliths in the urinary tract, the likelihood of the presence of struvite-type uroliths lead to favor acidification of the urine rather than a surgical approach to the obstructive site to elevate it. Based on the scar immediately over the bladder and the lack of scarring noted around the intraluminal pellet causing the urethral obstruction, the most likely trajectory of this pellet was intravesicular immediately after the animal was shot, with delayed migration to the urethra.

In this case, the owner subsequently acknowledged that the goat was most likely injured seven months prior after breaking out of a gate and entering the neighbor's garden. The timing of the temporal association with being shot at by the neighbor and the presentation for urethral obstruction in this case was approximately seven months. In the veterinary literature, there are several reports of delayed migration of ballistic foreign bodies in other species. In the case of a 22-caliber bullet causing a urethral obstruction in a three-year-old Border Collie, it was assumed that the shooting occurred approximately three months prior to presentation [4]. In a case involving a 4-year-old castrated male shorthair cat, dysuria was noted for approximately 8 weeks prior to presentation for diagnostic imaging where radiographs detected multiple metallic objects, presumed to be BB pellets (note: a “BB” pellet is the name for spherical metal projectiles approximately 4.3-4.4 mm in diameter) in the pelvis and near the pelvic urethra [9]. An additional feline case, an 8-year-old domestic shorthair, presented for stranguria and azotemia, and abdominal radiographs identified a ballistic foreign object in the bladder lumen [10]. This case was managed with the removal of the ballistic foreign body via cystotomy. In the human literature, a bullet lodged in the bladder wall of a 21-year-old male eventually migrated into the bladder and caused a urethral obstruction three years after the initial gunshot injury [5]. Upper urinary obstructions have been reported following gunshot in people. A ureteral obstruction secondary to a pellet has been noted in a 42year-old male five months after a gunshot [6]. Some of the reported upper urinary tract obstructions secondary to gunshot in the human literature occurred more closely to the shooting event, with case reports describing obstruction within one day, three days, and two weeks of the initial injury [11–13]. While immediate presentation of obstruction can occur, clinicians should be prepared to discuss the potential for delayed timelines of possible bullet migration that could develop into urethral obstructions in patients with intra-abdominal pellets in the periurinary and pelvic regions. The patient was discharged with the owners knowing the other ballistic pellet remained in the patient. The owners were made aware of the potential risk of migration of the second pellet for the goat in this case, but due to that pellet's more superficial nature and location, this risk of complications from migration was thought to be low.

Projectile injuries are not uncommon in the veterinary literature. In the present case, a 4.5 mm pellet was removed from the urethra of the patient. Such projectiles are usually fired from “pellet” guns, which are powered by a spring and/or compressed air or gas [14]. These guns are also more likely to be used by younger shooters than gunpowder-delivered projectiles [15]. Projectiles fired from these types of guns can achieve speeds as high as 304 meters per second, with the standard velocities ranging from 61 to 122 meters per second [15, 16]. While these guns are typically considered less dangerous than conventional gunpowder-delivered bullets, there are multiple cases in the literature of this type of projectile injuring small animals, and this case does highlight the potential for trauma from one of these guns to a large animal.

This case illustrates the importance of adhering closely to radiological principles and the complementarity of diagnostic imaging techniques. On the initial presentation of the patient, orthogonal radiographs were not immediately acquired. This practice is commonly used when evaluating urinary obstructed small ruminants. Considering the unusual presence of a ballistic foreign body in the perineum and the absence of radiopaque uroliths, retrieving orthogonal radiographs should have been obtained and might have streamlined the treatment process of the patient. However, it is also possible the pellet could not have been recognized as being in the urethra, as this is quite rare occurrence. In this particular case, the location of the pellet and its obstructive nature to urinary flow could only be fully determined and evaluated with the use of positive contrast urethrography and eventually with the use of ultrasound.

Ultrasound was essential in the presurgical phase. First, ultrasound allowed for the exact location of the pellet and confirmation that the pellet was surgically accessible once the patient was placed in surgical recumbency. Ultrasound also allowed for a reduced surgical incision as the exact location of the pellet was identified. Finally, the perioperative flushing of the urethra with concurrent visualization of the pellet with ultrasound confirmed the pellet was freely moveable in the urethra, which indicated there were no adhesions between the pellet and the urethra.

Currently, in the state of Tennessee, it does not appear to be legal to kill an animal on purpose, without the owner's consent, unless the animal was creating an immediate danger of death or serious injury (Tenn. Code § 39-14-205). In this case, the wether's owner informed the treatment team that their neighbor was trying to shoot at the goats with a perceived less-than-lethal projectile, as to scare the goats out of their (neighbor's) garden. It is also unknown at this time if this was the incident that led to the projectile injury to the goat in this case report. Ultimately, no charges were filed in this case.

Multiple drugs, in this case, were used in an extralabel fashion. These drugs included flunixin meglumine, meloxicam, procaine penicillin G, and the anesthetic agents. Flunixin and meloxicam were used as anti-inflammatory and analgesic agents, and currently no pain medications are labelled for use in goats in the United States [17–19]. While concern exists with increased potential adverse effects when multiple nonsteroidal anti-inflammatory drugs are administered to the same patient, flunixin was administered for a short time period, followed by meloxicam to limit overlap. Additionally, cases of initiating treatment with flunixin and then converting to meloxicam are reported without adverse effect in the caprine literature [20]. Withdrawal advice was obtained from the Food Animal Residue Avoidance Databank and communicated to the client at discharge.

In the presented case, a urethral obstruction secondary to the presumptive migration of a projectile pellet introduced to the bladder by gunshot seven months prior to presentation was diagnosed in a one-year-old wether. Diagnostic imaging included abdominal radiography, contrast urethrography, and perineal ultrasound. The case was surgically managed with a tube cystostomy and perineal urethrotomy. The importance of radiographic orthogonal views and the availability of multiple imaging modalities cannot be understated in this case and the diagnostic workup of urinary obstructions in goats, as identifying the precise location of an obstruction within the urinary tract may alter treatment plans and surgical approaches. Clinicians should consider the migration of foreign objects, such as projectiles, as a differential diagnosis for urethral obstruction in animals with evidence or a history of previous gunshot.

## Data Availability

All data is present within the manuscript.
